# Liposomal Doxorubicin, Vinblastine and Dacarbazine Plus Consolidation Radiotherapy of Residual Nodal Masses for Frontline Treatment in Older Adults With Advanced Stage Classic Hodgkin Lymphoma: Improved Outcome in a Multi‐Center Real‐Life Study

**DOI:** 10.1002/hon.70003

**Published:** 2024-11-17

**Authors:** M. Picardi, A. Vincenzi, C. Giordano, L. De Fazio, N. Pugliese, A. Scarpa, E. Vigliar, G. Troncone, D. Russo, M. Mascolo, G. Esposito, M. Prastaro, C. Santoro, R. Esposito, C. G. Tocchetti, C. Mainolfi, R. Fonti, S. Del Vecchio, M. Carchia, C. Quagliano, A. Salemme, V. Damiano, R. Bianco, F. Trastulli, F. Ronconi, M. Annunziata, F. Pane

**Affiliations:** ^1^ Department of Clinical Medicine and Surgery Federico II University Medical School Naples Italy; ^2^ Department of Public Health Federico II University Medical School Naples Naples Italy; ^3^ Department of Advanced Biomedical Sciences Federico II University Medical School Naples Italy; ^4^ Departments of Translational Medical Sciences Federico II University Medical School Naples Italy; ^5^ Hematology Unit Antonio Cardarelli Hospital of National Importance Naples Italy

**Keywords:** c‐HL, consolidation radiotherapy, elderly patients, MVD, non‐pegylated liposomal doxorubicin

## Abstract

In elderly patients with high‐risk classic Hodgkin lymphoma (c‐HL), we evaluated the impact of a new modality treatment without bleomycin, that is, liposomal doxorubicin (NPLD)‐based regimen plus consolidation radiotherapy of residual nodal masses (RNMs), on overall survival (OS) and progression free survival (PFS). In this retrospective study (2013–2023) conducted in tertiary hospitals in the bay of Naples (Italy), 50 older adults (median age, 69 years; range, 60–89) with advanced stage c‐HL received frontline treatment with MVD ± irradiation. MVD consisted of 25 mg/m^2^ of NPLD along with standard Vinblastine and Dacarbazine for a total of 6 cycles (twelve iv administrations, every 2 weeks) followed by radiation of RNMs with size ≥ 2.5 cm at computed tomography. Patients underwent MVD with a median dose intensity of 92%. At 2‐deoxy‐2[F‐18] fluoro‐D‐glucose positron emission tomography (FDG‐PET), 90% of patients (45/50 patients; one failed to perform final FDG‐PET due to early death) reached complete responses. Altogether, 17 patients (34%) received consolidation radiotherapy of RNMs with Deauville score ≥ 3. At 5‐year median follow‐up, the OS and PFS of the entire population were 87.5% (95% confidence interval [CI], 78.7–97.4) and 81.6% (95% CI, 71.4–93.2), respectively. Eleven patients (22%) experienced grade ≥ 3 adverse events, and 4 of them required hospitalization. Our data suggest that in older adults with high‐risk c‐HL NPLD‐driven strategy (without bleomycin) plus consolidation radiotherapy (if needed) may be a promising up‐front option, to test in phase II clinical trials for improving survival incidence.

## 1 Introduction

Patients aged ≥ 60 years represent approximately 20% of all cases of classic Hodgkin lymphoma (c‐HL) [1]. Clinically, these patients often present with negative prognostic factors such as advanced stage and B symptoms [2]; coupled with this are frequent comorbidities with poor Eastern Cooperative Group Performance Status (ECOG PS), which may affect the ability to administer curative‐intent combination chemotherapy at standard doses or in a timely fashion [1, 2]. The optimal therapy in this age group remains poorly defined. As a result, outcomes in older adults with c‐HL have historically been dismal [1, 2, 3]. ABVD (Adriamycin, Bleomycin, Vinblastine, Dacarbazine) regimen has been an option for elderly subset with c‐HL but may be associated with treatment toxicity and even mortality. These unfavorable events are especially related to conventional anthracycline and/or bleomycin [4, 5, 6, 7].

Myocet is doxorubicin encapsulated in a non‐pegylated liposomal membrane of phosphatidylcholine and cholesterol [8]. Non‐pegylated liposomal doxorubicin (NPLD) was initially used in the treatment of patients affected by breast cancer, and a peculiar characteristic emerged for this agent [9]. Liposome formulations spare the healthy tissues characterized by tight endothelial capillary junctions, like the heart muscle, from the direct cytotoxic drug effect [8, 9]. In a phase II study, NPLD (Myocet) was investigated together with Bleomycin, Vinblastine, Dacarbazine (MBVD) for the frontline therapy of cardiopathic patients with c‐HL, showing about 40% treatment discontinuation rate mostly due to severe neutropenia and/or pneumonitis [10]. However, in vitro studies prove some pharmacokinetic and pharmacodynamic advantages of liposomal doxorubicin [11]. It rapidly accumulates at high‐levels within the reticulo‐endothelial system of spleen, liver, lung, and bone. Liposomal doxorubicin acts as a slow‐release reservoir with prolonged powerful tumoricidal effects specifically inside the neoplastic tissue, that is, within tumor‐associated macrophages (TAMs) of the lymphadenopathy microenvironment [12]. In real‐life, these effects might be perceived as a great benefit especially in those patients with more aggressive disease. In elderly patients, there is a relevant frequency of high‐tumor burden with nodal masses and extra‐nodal involvements [13]. An emerging report shows that in older adults TAMs are frequently ≥ 5% staining at immunohistochemical analysis on biopsy specimens of lymph nodes [14]. A specific strategy [15] is routinely used in three tertiary hospitals in the bay of Naples (Italy) for elderly patients with untreated c‐HL in stage III/IV. It consists in the up‐front administration of a new cytotoxic agent regimen without bleomycin and with NPLD, so called MVD, that is, Myocet, Vinblastine, Dacarbazine. Afterward, consolidation radiation is given on post‐chemotherapy residual nodal masses (RNMs), as already described [16, 17].

Herein, we report a multicentric real‐life experience regarding the outcome of patients aged ≥ 60 years with advanced stage c‐HL undergoing frontline treatment with MVD ± irradiation.

## 2 Patients and Methods

### 2.1 Study Design

This was a retrospective, multi‐center study using the medical records or local database of the Hematology Unit of the Federico II University of Naples (Italy), Oncology Unit of the Federico II University of Naples (Italy), and Hematology Unit of the Antonio Cardarelli Hospital of national importance of Naples (Italy). These three clinical units had in common the same local ethics committee, and similar internal guidelines for the management of patients with HL [18, 19]. Patients aged ≥ 60 years with previously untreated, biopsy‐proven c‐HL [20, 21, 22, 23] consecutively referred to the clinical Units above reported for curative‐intent antineoplastic treatment from 1 January 2013 to 1 January 2023 were eligible (Supporting Information S1: Methods).

The primary endpoint was the activity of liposomal doxorubicin‐based frontline strategy (without bleomycin) combined with consolidation radiotherapy of RNMs (MVD ± irradiation) in terms of overall survival (OS) and progression free survival (PFS) at 5‐year median follow‐up. Secondary endpoints were the rates of response at end‐of‐treatment (EoT) by using 2‐deoxy‐2[F‐18] fluoro‐D‐glucose positron emission tomography (FDG‐PET), toxicity (including cardiologic side‐effects), and feasibility. Noteworthy, the cardiologic toxicity profile was established by using the echocardiography assessment of global systolic longitudinal myocardial strain (GLS), as well as left ventricular ejection fraction (LVEF) [6].

### 2.2 MVD ± Irradiation: Treatment Plan

**FIGURE 1:**
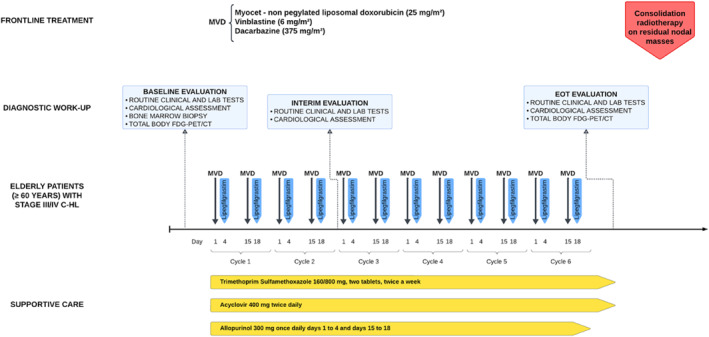
Drug doses, schedule, and treatment administration details of frontline MVD regimen ± irradiation. Dose‐intensity and dose‐dense of non‐pegylated liposomal doxorubicin in cycles 1‐6 for MVD, and diagnostic work‐up and vigorous support treatments are also shown. FDG‐PET/CT, 2‐deoxy‐2[F‐18] fluoro‐D‐glucose positron emission tomography/computed tomography; MVD, Myocet, Vinblastine, Dacarbazine.

The treatment regimen is shown in detail in Figure 1. The schedule consisted of six MVD cycles, that is, 1‐day outpatient intravenous infusions of Myocet at a dose of 25 mg/m^2^, plus vinblastine 6 mg/m^2^, and dacarbazine 375 mg/m^2^ on days 1 and 15 of each cycle every month, for 12 administrations. Planned cumulative dose‐intensity of NPLD was 300 mg/m^2^. For those cases with baseline large nodal masses (LNMs), defined as systemic adenopathy with the largest diameter > 5 cm, consolidation radiotherapy (30 Gy) with a linear accelerator was routinely given on residual bulky area, that is, containing RNMs, that is, post‐chemotherapy nodes of size ≥ 2.5 cm at CT scans, regardless of Deauville scale (DS) 5‐point scoring system results at FDG‐PET assessments (except for patients with new FDG‐avid foci consistent with progressive disease, who were scheduled to systemic salvage regimen), as already reported [16, 17]. Daily fraction size was about 1.6 Gy five times a week (for a total of 4 weeks) [7, 16, 17, 24].

### 2.3 Inclusion Criteria

We included in the final analysis patients aged ≥ 60 years with histologic diagnosis of c‐HL [20, 21, 22, 23] receiving as frontline therapy at least one MVD course ± irradiation. Other criteria of inclusion were Ann Arbor stages III and IV, ECOG PS 0–3, LVEF ≥ 50% with any result of GLS at echocardiographic assessment [5, 6, 7], and human immunodeficiency virus negativity at baseline. Patients were excluded from the analysis if they had concomitant major illnesses at baseline (Supporting Information S1: Methods).

### 2.4 Supportive Care

Long‐acting recombinant granulocyte‐colony stimulating factor, that is, lipegfilgrastim (a glycopegylated modification of filgrastim: Lonquex) was routinely administered subcutaneously on days 4 and 18 of every 1‐month cycle of MVD. In addition, antimicrobial drugs were routinely administered for each patient (Figure 1). Other supportive medications were given at physician discretion (Supporting Information S1: Methods) [25].

### 2.5 Clinical Evaluations and Imaging Assessments

Physical examination and bone marrow biopsy were performed at baseline, and then at physician's discretion. Routine blood laboratory test monitoring was performed before every cycle of chemotherapy for each patient.

FDG‐PET examinations were conducted at staging, EoT and thereafter every 3 months for the first 2 years, and every 6 months for the next 3 years, as previously described [26, 27, 28, 29]. FDG‐PET results were reported according to the DS score using visual assessment followed by quantitative verification as already described (Supporting Information S1: Methods) [18, 19].

Patients routinely underwent a full cardiologic examination: 2D echocardiography and speckle tracking echocardiography (STE) at baseline, interim, EoT and within 6 months from the end of all antineoplastic treatments, as already reported [5, 6, 7].

### 2.6 Statistical Analysis

More details for efficacy and safety evaluations were reported in Supporting Information S1 (Methods). The efficacy evaluations were performed in the entire cohort, and then in the cohort of patients who received chemotherapy alone (MVD) and in the cohort of patients who received combined modality treatment (MVD + radiation). All safety evaluations were performed in patients who received at least one course of MVD.

Cox regression analysis was used to estimate the hazard ratio (HR) and the 95% confidence interval (CI) for the treatment effect on OS and PFS. Differences between groups were tested by the log‐rank test, Mann Whitney test, *χ* ^2^ and student‐*T* test. The *p* value for statistical significance was set at 0.05 for all evaluations. Statistical analysis was performed using R software (version 3.6.3).

## 3 Results

### 3.1 Patient Characteristics

**FIGURE 2:**
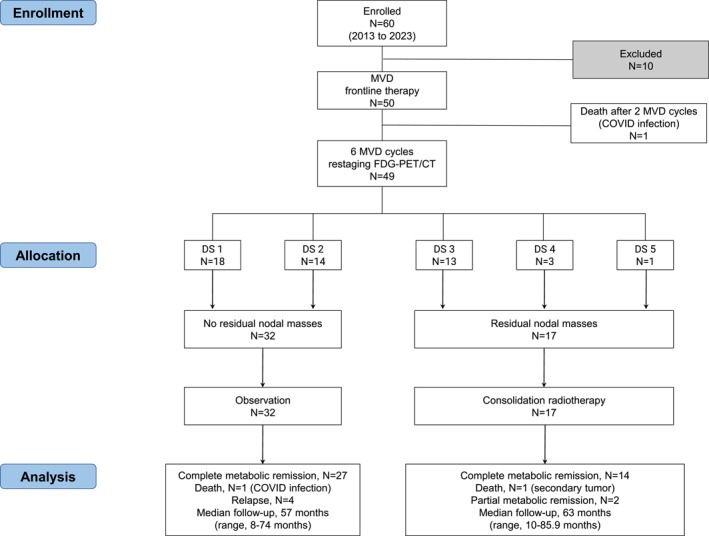
Flow of participants. DS, Deauville scale scoring system; FDG‐PET/CT, 2‐deoxy‐2[F‐18] fluoro‐D‐glucose positron emission tomography/computed tomography; MVD, myocet, vinblastine, dacarbazine.

On initial review of the medical records or database of the tertiary hospitals in the bay of Naples (Italy), 60 consecutive patients aged ≥ 60 years with newly diagnosed advanced stage c‐HL who were about to receive curative‐intent anticancer therapy were identified from January 2013 to January 2023, with follow‐up to December 2023. Ten patients were excluded (Supporting Information S1: Results). The remaining 50 patients, who received at least one MVD course ± irradiation, were included in the final analysis. A diagram in Figure 2 summarizes the flow of patients through the study: the entire series of patients (*n* = 50), and 33 patients receiving MVD (chemotherapy alone cohort) and 17 patients receiving MVD + radiation (combined modality treatment cohort). The main characteristics of the 50 evaluable patients are reported in Table 1. The median age was 69 years (range, 60–89 years), with the majority (27, 54%) of patients aged between 60 and 69 years, 17 (34%) patients aged between 70 and 79, and a minority of patients older ≥ 80 years (6, 12%). Twenty‐five patients (50%) had LNMs. The invasion of spleen, bone, lung, and/or liver was found in 7 (14%), 11 (22%), 9 (18%), and 2 (4%) patients, respectively. As comorbidities, over two‐third of patients (80%) had at least two of the traditional cardiac risk factors (Supporting Information S1: Results).

**TABLE 1:** Patients' characteristics at baseline.

Characteristics	Total series MVD ± irradiation	Chemotherapy alone MVD	CMT MVD + radiation
Number of patients	50	33	17
Male sex	27 (54%)	16 (48%)	11 (64%)
Age, median (range) years	69 (60–89)	69 (60–86)	74 (61–89)
60–69 years	27 (54%)	20 (60%)	7 (41%)
70–79 years	17 (34%)	9 (27%)	8 (47%)
≥ 80 years	6 (12%)	4 (12%)	2 (12%)
c‐HL histological subtype			
NS	34 (68%)	21 (63%)	13 (76%)
MC	15 (30%)	12 (36%)	3 (17%)
LR	1 (2%)	0 (0%)	1 (7%)
ECOG‐PS 0–2	31 (62%)	20 (61%)	11 (64%)
ECOG‐PS 3	19 (38%)	13 (39%)	6 (36%)
Ann Arbor stage			
III	28 (56%)	20 (60%)	8 (47%)
IV	22 (44%)	13 (40%)	9 (53%)
B symptoms	25 (50%)	15 (45%)	10 (58%)
Number of nodal sites involved median (range)	8 (3–11)	8 (3–11)	7 (3–11)
Patients with < 4 sites	20 (40%)	14 (42%)	6 (35%)
Patients with ≥ 4 sites	30 (60%)	19 (57%)	11 (65%)
Large nodal masses			
Number of patients	25 (50%)	8 (24%)	17 (100%)
Median size in cm (range)	6.5 (5.3–8.5)	6.0 (5.3–6.5)	7.5 (5.5–8.5)
Extranodal sites			
Spleen	7 (14%)	4 (12%)	3 (17%)
Bone	11 (22%)	7 (21%)	4 (23%)
Lung	9 (18%)	5 (15%)	4 (23%)
Liver	2 (4%)	1 (3%)	1 (6%)
IPS < 3	28 (56%)	20 (61%)	8 (47%)
IPS ≥ 3	22 (44%)	13 (39%)	9 (53%)
Cardiac comorbidities	30 (60%)	24 (72%)	6 (35%)
Pulmonary comorbidities	10 (20%)	8 (24%)	2 (11%)

*Note:* Values are *n* (%) unless otherwise specified. Chemotherapy alone cohort received MVD (Myocet, Vinblastine, Dacarbazine); CMT (Combined Modality Treatment) cohort received MVD + consolidation radiotherapy on residual nodal masses. Cardiac comorbidities: hypertension, obesity, tobacco use, diabetes mellitus, hyperlipidemia, history of heart disease, coronary artery disease, atrial fibrillation, heart transplanted for cardiomyopathies. Pulmonary comorbidities: chronic obstructive pulmonary disease.

Abbreviations: c‐HL, classic Hodgkin lymphoma; ECOG‐PS, eastern cooperative group performance status (ECOG 3: patients were capable of limited self‐care, confined to bed or chair more than 50% of time); IPS, international prognostic score, including age, sex, stage, hemoglobin level, albumin level, lymphocyte blood count and white blood cell count; Large nodal mass, defined as lymph node mass with long axis > 5 cm; LR, lymphocyte‐rich subtype; MC, mixed cellularity subtype; NS, nodular sclerosis subtype.

All patients received supportive care, as described above.

### 3.2 MVD Regimen: Final Responses

Overall, 49 out of 50 patients (98%) underwent FDG‐PET examinations after six MVD cycles, whereas one patient did not for toxicity reasons. Except in this case, all patients were assessable for the metabolic response. Altogether, the analysis of FDG‐PET scans assigned a DS score as follows: DS score 1 to 18 patients, DS score 2 to 14 patients, DS score 3 to 13 patients, DS score 4 to three patients, and DS score 5 to one patient (Figure 2). Thus, 45 out of 50 patients had FDG‐PET scans with DS score ≤ 3 at chemotherapy completion and before consolidation radiotherapy start (if needed, as reported above).

### 3.3 MVD Regimen: Feasibility, Treatment Delivery and Dose‐Intensity

Thus, 49 out of 50 patients (98%) completed six MVD courses; the remaining patient died for infection (COVID‐19) after the second cycle of MVD and was recorded as a failure of the therapeutic strategy and included in the analysis of all efficacy evaluations (in the chemotherapy alone cohort). Regarding dose‐intensity of planned MVD treatment, 46 patients received full‐dose (100%), three patients received a dose‐intensity between 85% and 99%, while only one patient (2%; 1/50) received a dose reduction of > 15%. Therefore, the feasibility endpoint (≤ 10% of patients receiving < 85% of the planned dose) was reached. The mean dose‐intensity for the overall patient population (*n* = 50 cases) undergoing MVD was 92%, with a range of 33%–100%. The median duration of MVD was 190 days (range, 58–190 days) as the expected duration of 190 days.

### 3.4 Consolidation Radiotherapy

After induction MVD treatment, altogether 17 patients (34%) constituted the combined modality treatment cohort receiving consolidation radiotherapy. As shown in Table 1, the patients undergoing combined modality treatment were of older age (median age, 74 years), more extensive disease (stage IV, 53% of cases), more often IPS ≥ 3 (53% of cases), and were more frequently with LNMs and characterized with greater size as compared with patients undergoing chemotherapy alone. Table 2 shows radiotherapy details. The field of irradiation included the mediastinal site (*n* = 3 cases), and extra‐mediastinal site (*n* = 14 cases). Consolidation radiotherapy was given in all cases on RNMs of size ≥ 2.5 cm (regardless of DS 5‐point scoring system at FDG‐PET assessment): long axis diameter median was 4 cm (range, 2.5–4.5 cm) at CT scans. Before irradiation, the analysis of FDG‐PET scans of the 17 cases assigned a DS as follows: DS score 3 to 13 patients, DS score 4 to three patients, and DS score 5 to one patient (Figure 2). As compared to the baseline PET scans, no new site of disease was found in the 17 patients at FDG‐PET assessments.

**TABLE 2:** Patients treated with consolidation radiotherapy on residual nodal masses following MVD regimen (CMT cohort).

Patient	Age (years)	Ann Arbor stage	LNM sites	MVD cycles	LNM size (cm): Pre‐MVD/post‐ MVD	Deauville score post‐MVD FDG‐ PET	Irradiated sites	Radiotherapy dose (Gy)	Outcome after CMT	Salvage regimen	Months of follow‐up
#1	61,7	IV	Neck	6	7,0/4,0	3	1	30	Alive, in CMR	NA	63
#2	68	IV	Neck, axillary	6	7,5/3,9	3	2	30	Alive, in CMR	NA	37
#3	74,5	IV	Inguinal	6	6,5/3,5	4	1	30	Alive, in CMR	NA	77
#4	61,5	IV	Mediastinum	6	7,5/4,0	3	1	30	Alive, in CMR	NA	6
#5	68,8	IV	Neck	6	7,5/4,5	4	1	30	Alive, in PMR	Nivo	64
#6	68,8	III	Neck	6	7,5/3,0	3	1	30	Alive, in CMR	NA	44
#7	70,5	III	Inguinal	6	7,5/2,5	3	1	30	Alive, in CMR	NA	86
#8	78,7	IV	Mediastinum	6	7,5/4,5	5	1	30	Alive, in PMR	Bv	60
#9	88,7	IV	Mediastinum	6	5,5/2,5	4	1	30	Alive, in CMR	NA	71
#10	68,6	III	Inguinal	6	7,5/4,5	3	1	30	Alive, in CMR	NA	67
#11	74,9	III	Neck	6	7,5/4,5	3	1	30	Alive, in CMR	NA	63
#12	76,4	III	Inguinal	6	5,5/3,0	3	1	30	Alive, in CMR	NA	52
#13	74	III	Neck	6	7,5/4,5	3	1	30	Alive, in CMR	NA	54
#14	63,6	III	Neck	6	5,5/2,5	3	1	30	Alive, in CMR	NA	75
#15	74	IV	Bilateral inguinal	6	5,5/2,5	3	2	30	Alive, in CMR	NA	11
#16	67,2	IV	Bilateral neck	6	6,5/3,5	3	2	30	Alive, in CMR	NA	7,3
#17	83,1	III	Inguinal	6	6,5/4,0	3	1	30	Dead	NA	16

Abbreviations: Bv, brentuximab vedotin; cm, centimeters; CMR, Complete Metabolic Remission; CMT, combined modality treatment (MVD + radiation); FDG‐PET, Fluor‐Deoxy‐Glucose Positron Emission Tomography; Gy, gray; LNM, Large Nodal Mass; MVD, Myocet, Vinblastine, Dacarbazine; NA, not applicable; Nivo, nivolumab; Stage III, nodal masses on both sides of the diaphragm; Stage IV, disseminated or nodular involvement of one or more extralymphatic organs, including any involvement of the liver, bone marrow or lungs.

At the time of EoT FDG‐PET (after MVD ± irradiation, as scheduled), 47 patients obtained complete metabolic remission thus with a complete response rate of 94%, two patients resulted with partial metabolic remission and one patient died from acute infectious toxicity (during induction therapy).

### 3.5 Outcome of c‐HL in Elderly Patients

The median follow‐up for the entire series of 50 elderly patients with advanced stage c‐HL undergoing MVD ± irradiation was 60 months with a range of 2–86 months. Altogether, there were six patients with persistent/relapsed lymphoma during the analyzed period. Specifically, two patients had residual disease after MVD + radiation with partial metabolic response at FDG‐PET assessments, and four patients in the chemotherapy alone cohort had relapsed disease at a median follow‐up of 13 months (range, 11–15 months). As salvage treatment, four cases received single agent therapy with nivolumab, and two cases received single agent therapy with brentuximab vedotin (BV). Altogether, there were six fatal events during the analyzed period. Three patients in the chemotherapy alone cohort died for disease progression after relapse, two cases in the chemotherapy alone cohort died for infection (one COVID‐19 during induction therapy, and one COVID‐19 during post‐treatment follow‐up) and one case in the combined modality treatment cohort died due to secondary tumor (gastric adenocarcinoma).

### 3.6 Overall Efficacy Results and Subgroup Analysis

**FIGURE 3:**
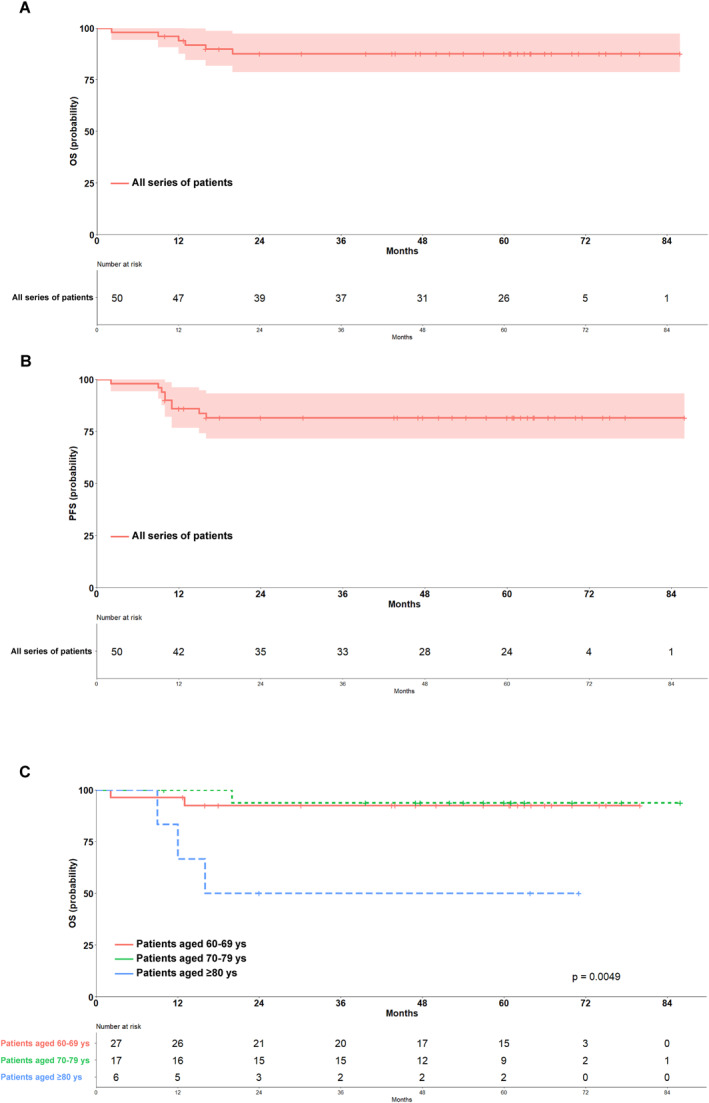
(A–C) Overall survival (OS) and Progression free survival (PFS). Kaplan Meier curves of 60 months OS and PFS (A and B, respectively) of 50 elderly patients with advanced stage classic Hodgkin lymphoma (c HL) who received the liposomal doxorubicin based (without bleomycin) frontline strategy ± irradiation. OS for elderly patients with c HL (n= 50) according to age stratification: 60‐69 years (n= 27) vs. 70‐79 years (n= 17) vs. ≥ 80 years (n= 6) (C). Figures also show the number of events and the number at risk during follow up.

The main efficacy results of the study treatments in the entire population, in the cohort of chemotherapy alone and in the cohort of combined modality treatment, are reported in Table 3. In the entire series, the 5‐year OS was 87.5% (95% CI, 78.7–97.4) and the 5‐year PFS was 81.6% (95% CI, 71.4–93.2) (Figures 3A and 3B). In the chemotherapy alone cohort, the 5‐year OS was 84.1% (95% CI, 72.1–97.9) and the 5‐year PFS was 81.5% (95% CI, 69.1–96.0) (Figures S1 and S2). In the combined modality treatment cohort, the 5‐year OS was 94.1% (95% CI, 83.6–100) and the 5‐year PFS was 81.9% (95% CI, 65.3–100) (Figures S1 and S2).

**TABLE 3:** Main efficacy results in the entire population, and in the cohort of patients receiving chemotherapy alone and combined modality treatment (chemotherapy and radiotherapy) as frontline strategy for advanced‐stage classic Hodgkin lymphoma in elderly patients.

Outcome	All patients (MVD ± irradiation) *n* = 50	Chemotherapy alone (no residual nodal masses post‐MVD)^a^ *n* = 33	Combined modality treatment (residual nodal masses post‐MVD)^a^ *n* = 17
ORR^b^	98% (49/50)	97% (32/33)	100% (17/17)
CRR^b^	94% (47/50)	97% (32/33)	88.2% (15/17)
PRR^b^	4% (2/50)	0 (0/33)	11.8% (2/17)
60‐month PFS	81.6% (95% CI, 71.4–93.2)	81.5% (95% CI, 69.1–96.0)	81.9 (95% CI, 65.3–100)
60‐month OS	87.5% (95% CI, 78.7–97.4)	84.1% (95% CI, 72.1–97.9)	94.1% (95% CI, 83.6–100)

Abbreviations: CI, confidence interval; CRR, complete response rate; MVD, Myocet, Vinblastine, Dacarbazine; ORR, overall response rate; OS, overall survival; PFS, progression free survival; PRR, partial response rate.

^a^ Residual nodal mass: residual bulky area, containing post‐chemotherapy nodes of size ≥ 2.5 cm at CT scans with DS 5‐point scoring system of ≥ 3 and no new site of disease at FDG‐PET assessments, as already reported [16, 17].

^b^ One patient died after two cycles of MVD without performing FDG‐PET scans of revaluation.

Univariable analyses according to the pre‐specified subgroups showed that the patients with 60‐ to 69‐year and 70‐ to 79‐year were significantly associated with better OS as compared to the patients with ≥ 80 years (*p* = 0.0049), as well as the absence of B symptoms was associated with better OS (100% [95% CI, 100–100]) as compared to the presence of B symptoms (79.3% [95% CI, 65.8–95.6]) (*p* = 0.037). Cox regression analyses confirmed that OS of patients aged 60–69 years and 70–79 years (5‐year OS: 92.4% [95% CI, 82.9–100] and 93.7% [95% CI, 82.6–100], respectively) was better than that of patients aged ≥ 80 years (5 years OS: 50% [95% CI, 22.5–100]) (HR of 3.14 [95% CI: 1.061–9.279]; *p* = 0.0049) (Figure 3C). Overall, by age group, the 5‐year PFS in those patients aged 60 to 69, 70 to 79, and ≥ 80 years was 85% (95% CI, 72.5–99.7), 87.8% (95% CI, 73.4–100), and 50% (95% CI, 22.5–100), respectively (*p* = 0.078).

### 3.7 Toxicity

Table 4 reports the major adverse events related to the study treatments.

**TABLE 4:** Safety results in the entire series of patients, stratified according to the grades of CTCAE.

Variable	Total, *n* (%)	Grade < 3, *n* (%)	Grade ≥ 3, *n* (%)
Num. of patients	36 (72)	25 (50)	11 (22)
Hematological toxicity			
Anemia	7 (14)	4 (8)	3 (6)
Neutropenia	5 (5)	4 (8)	1 (2)
Thrombocytopenia	5 (5)	5 (10)	0
Extra‐hematological toxicity			
Febrile neutropenia	1 (2)	0	1 (2)
Upper respiratory tract infections	4 (8)	3 (6)	1 (2)
Alveolitis	2 (4)^a^	0	2 (4)^a^
Nausea	16 (32)	16 (32)	0
Diarrhea	15 (30)	15 (30)	0
Constipation	20 (40)	20 (40)	0
Dizziness	19 (38)	19 (38)	0
Hepatitis	1 (2)	0	1 (2)
Fatigue	25 (50)	25 (50)	0
Rash	2 (4)	2 (4)	0
Cardiovascular toxicity			
Deep venous thrombosis	2 (4)	2 (4)	0
Atrial fibrillation	2 (4)	0	2 (4)
Left ventricular systolic dysfunction	5 (10)	5 (10)	0

*Note:* All side effects possibly related to treatment schedule were reported according to the common terminology criteria for adverse events (CTCAE).

^a^ These two patients died after two cycles of chemotherapy and 2 months after the end of chemotherapy, respectively, for COVID‐19 infection.

#### 3.7.1 Non‐Cardiologic Toxicity

Regarding hematological toxicity, a total of 3 (6%) patients suffered from anemia of grade (G) 3, and one patient (2%) suffered from at least one neutropenic event of G3.

Infections occurred in one patient (2%) as febrile neutropenia of G3, in one patient (2%) as upper respiratory tract infection of G3, and in two patients (4%) as alveolitis (COVID‐19) of G5 for both.

One patient (2%) suffered from G4 gastrointestinal toxicity event (hepatitis).

#### 3.7.2 Cardiologic Toxicity

A complete echocardiographic evaluation (including measurements of GLS and LVEF performed at baseline, interim, EoT and six months later) was available for 45 patients.

**FIGURE 4:**
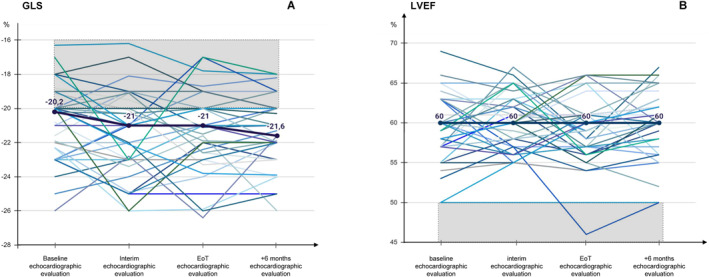
(A, B) Percentage variations in global systolic longitudinal myocardial strain (GLS) (A) and left ventricular ejection fraction (LVEF) (B) and throughout treatment up to 6‐month after completion of study treatments expressed in individual values. Shaded areas represent abnormal values of echocardiographic measurements. The bold curve represents the median values at all time points.

At baseline (chemotherapy start), there were 7 patients (15%) with measurements of GLS less than −20% (they had LVEF measurements ≥ 50%); the echocardiographic assessment showed median result of GLS of −20% and median result of LVEF of 60%. At the interim assessment, the median result of GLS was −21% and the median result of LVEF was 60%. At EoT assessment, the median result of GLS was −21% and the median result of LVEF was 59%. At 6‐month follow‐up, the median result of GLS was −21% and the median result of LVEF was 60%. For the majority of patients, there were very small changes, that is, < 10% points reductions in median values of GLS and LVEF at interim, EoT and 6‐month follow‐up, when they were compared with the median values at baseline. Only four measurements (in four patients) of GLS resulted worsened with ≥ 15% points reduction (Figure 4A) as compared to baseline, and only 6 measurements (in six patients) of LVEF resulted worsened with ≥ 10% points reduction (Figure 4B) as compared to baseline.

A total of 2 (4%) patients presented relapse of atrial fibrillation of G3, but prompt initiation of medical treatment led to complete reversal of cardiac abnormality.

Thus, only one (2%) out of 50 patients discontinued study treatment definitively due to extra‐cardiac toxic events of G5 (as above reported). Except for four cases, none of the remaining patients required hospitalization to manage treatment‐related adverse events.

## 4 Discussion

About one‐half of patients aged ≥ 60 years with c‐HL with extensive disease does not benefit from up‐front therapy with ABVD regimen [3, 4, 13]. This is a relevant issue in real‐life because in this age setting c‐HL is more frequently diagnosed in advanced stage than in limited stage [1, 2, 13]. According to up‐dates from scientific literature, alternative therapeutic strategies include the administration of selectively active agent‐based new regimens. Several controlled studies have been published with contradictory results [30, 31, 32]. For instance, in the phase III Echelon‐1 trial, up‐front BV in combination with Adriamycin, Vinblastine, Dacarbazine (AVD) in stage III/IV c‐HL demonstrated in patients aged < 60 years a 6‐year PFS and OS of 85% and 97%, respectively [30]. However, in this trial the rates of PFS and OS in the older subgroup were 66% and 74%, respectively. In a phase II trial, in untreated patients aged 60 years or older with stage II‐IV c‐HL, a regimen of BV sequentially administered before and after AVD showed 2‐year PFS and OS of 84% and 93%, respectively [31]. However, in this trial 42% of patients experienced grade 3 to 4 adverse events and consequently treatment discontinuations (mostly for febrile neutropenia and pneumonia). A phase III randomized trial (SWOG S1826) examining the frontline use of nivolumab with AVD (N‐AVD) in patients with c‐HL with extensive disease is ongoing (NCT03907488), and preliminary results show improving of PFS in patients treated with N‐AVD as compared to BV‐AVD [32]. However, the follow‐up of the N‐AVD sub‐group including patients aged ≥ 60 years is too short. More insight into the safety and efficacy of this combination for older patients will likely be provided in the following years. Thus, all these approaches are not routinely employed in the elderly subset because they have not been clearly proven effective, safe, and/or economically advantageous [33].

Our real‐life study, including a sufficiently large series of elderly patients with advanced stage c‐HL homogeneously treated with a risk‐adapted strategy, that is, chemotherapy alone in absence of RNMs and combined modality treatment in presence of RNMs, provides enough evidence of the efficacy of liposomal doxorubicin‐based (without bleomycin) frontline therapy plus radiation (if needed), that is, six MVD cycles followed by consolidation radiotherapy only focally on lymphadenopathies with size ≥ 2.5 cm in the initial bulky area, as reflected by a substantial increase in the number of survived patients in complete metabolic response without further antineoplastic therapy. At 5‐year median follow‐up, the OS rate was 87.5% versus 64% of the pooled summary OS rate of the literature following ABVD strategy in a similar setting of patients [1, 2, 3, 13, 34, 35] and the PFS rate was 81.7% versus 54% of the pooled summary PFS rate of the literature following ABVD strategy in a similar setting of patients [1, 2, 3, 13, 34, 35]. These results were considered of very important clinical interest by us due to the fact that there was an absolute improvement of at least 24% points of outcome. However, we admit that the comparison with the figures of standard approaches was approximate, due to personal extrapolations by the authors based on the features available in each report.

Three main findings of the study require attention, because they may explain the success of therapeutic strategy. First, by the up‐front administration of MVD regimen we avoided from the beginning exposure to both bleomycin sulfate and doxorubicin hydrochloride thus drastically reducing the risk of morbidity and mortality related to the two drugs [4, 5, 6, 13, 36]. Consistent with several publications, the use of bleomycin in elderly patients appears to be prohibitive: bleomycin lung toxicity is frequent and is diagnosed in up to 18% of older patients receiving this compound [4, 13]. Noteworthy, in about 40% of these patients, bleomycin lung toxicity is judged to be severe and potentially life‐threatening. The rate of deaths due to bleomycin‐related pulmonary damage ranges between 4% and 24% [4]. Conventional anthracycline can induce cardiotoxicity, usually by means of irreversible damage to cardiomyocytes that can manifest with left ventricle remodeling, dilation, and eventually heart failure with cardiomyocyte apoptosis and necrosis especially in the older subset [5, 6, 7, 36]. A growing number of reports underlines an important rate of long‐term fatal events due to heart damage related to conventional anthracycline‐based regimens, ranging between 6% and 15% [5, 6, 7, 36]. Second, in our series of older adults liposomal doxorubicin was used at standard doses of 25 mg/m^2^ despite several cardiac risk factors in the majority of them. Myocet has some pharmacokinetic and pharmacodynamic advantages in terms of safety and efficacy [9, 11, 12, 14, 15]. Preclinical studies have shown that delivery of liposomal doxorubicin is high through the disrupted capillaries bed of the tumor tissues, while both peak and overall concentrations of liposomal doxorubicin are reduced by 30%–40% in myocardial tissue [11, 12]. This diminished myocardial exposure resulted in a significant reduction of both functional and histological cardiac toxicity [10]. On the other hand, the accumulation of therapeutic liposomal nanoparticles inside tumor microenvironment theoretically enhances the susceptibility to chemotherapy and/or radiotherapy of cancer cells, by hampering pro‐tumor activities of CD68‐positive TAMs [14, 15]. The complete administration of six MVD cycles was accompanied by a rate of complete hematological responses of 90% (45/50 patients). In view of a non‐complete shrinkage (a relative reduction of about less than 50% of the maximal long axis diameter) of lymph nodes at initial mass sites, 17 of the 50 (34%) patients were given localized irradiation in fields including metabolically active residual tissue (Figure 2): in 13 cases with FDG uptake > mediastinum but ≤ liver (DS 3 scores at PET scans) and in 4 cases with FDG uptake moderately or markedly > liver (three in the category of DS 4 scores and one in the category of DS 5 score at PET scans). In this small cohort of patients protected by radiotherapy, in spite of the presence of several unfavorable prognostic factors at baseline, such as older age, and high‐tumor burden with more aggressive disease, at a median follow‐up of 5 years, 16 out of 17 patients (94%) were alive, and 14 out of 17 (82%) were in complete hematological remission status without further antineoplastic treatment (Figures S1 and S2). However, recent literature shows that radiation in such instances does not add a significant increase in PFS [37]. Finally, advances in supportive care is another factor that may explain the success of our therapeutic strategy. A robust primary prophylaxis with broad‐spectrum anti‐infectious support drug treatment, including long‐acting recombinant granulocyte‐colony stimulating factor, that is, lipegfilgrastim, and strict clinical and laboratory monitoring, is strongly recommended in this setting of elderly patients with c‐HL undergoing curative‐intent chemotherapy [38].

In our study, the anti‐cancer treatment was well tolerated. Overall, the rate of the toxicity of *G* ≥ 3 was 22%. There were 11 adverse events (in a total of 11 patients) of *G* ≥ 3: only two led to death (18%, 2/11), the other 9 events were all reversible with medical support, without requiring hospitalization in about 80% of cases. The dosage of Myocet in the MVD scheme was well within the ceiling dose of 785 mg/m^2^ (the median lifetime dose reported for NPLD at the onset of cardiotoxicity). Advanced echocardiographic techniques systematically performed by expert echocardiographers (for exploring subclinical signs of impaired ventricular function, i.e., strain rate imaging with measures of global radial and circumferential strain) [39] documented a preservation of myocardial ventricular function in most cases until 6‐month follow‐up after therapy.

Our study has some limitations. First, this was a retrospective study. This is a potential bias that could explain the extremely favorable outcome compared to the known literature. Second, a cost issue could be raised since up‐front therapy with liposomal doxorubicin is more expensive than conventional anthracycline. However, our strategy relevantly reduces the rate of patients potentially not cured with intent antineoplastic therapy. In fact, we treated a vast majority of patients, particularly with several cardiac comorbidities, using NPLD‐based cytotoxic agent regimen at standard doses and in a timely fashion. Third, our irradiation of the LNM site could be seen as not consistent with modern therapeutic approaches. However, consolidation radiotherapy has been incorporated into frontline treatment with good preliminary results [35]; in the last decade, radiotherapy dose and volumes have significantly decreased, thereby decreasing toxicity risks [40]. According to the European Society for Medical Oncology clinical practice guidelines for diagnosis, treatment and follow‐up of HL, the question of whether consolidation radiotherapy can be safely omitted in patients with advanced HL who have RNMs at the end of chemotherapy has not yet been definitively answered [41]. In our experience, based on the results of controlled clinical trials [16, 17], as well as in the experience of the authors of National Comprehensive Cancer Network clinical practice guidelines in oncology [42], the addition of radiotherapy is suggested to residual lymphadenopathies with FDG uptake of DS ≤ 3 scores in initial bulky areas or selected PET + sites. Finally, our strategy proves to be less effective for relatively older patients with age ≥ 80 years, regarding both OS and PFS.

In conclusion, for primary therapy, anthracycline‐based chemotherapy platforms are associated with the most robust outcomes for elderly HL patients [34, 35]. Our multi‐center, non‐controlled, real‐life study conducted in a high‐risk setting of elderly (age ≥ 60 years) patients with c‐HL presents convincing evidence that up‐front treatments with six cycles of Myocet, Vinblastine and Dacarbazione followed by irradiation of RNMs are a “proof of concept” for testing them in larger multicenter phase II clinical trial.

## Conflicts of Interest

The authors declare no conflicts of interest.

### Peer Review

The peer review history for this article is available at https://www.webofscience.com/api/gateway/wos/peer-review/10.1002/hon.70003.

## Supporting information

Supporting Information S1

Figure S1

Figure S2

## Data Availability

The data that support the findings of this study are available on request from the corresponding author. The data are not publicly available due to privacy or ethical restrictions.
