# Supplementary material for: Liposomal Doxorubicin, Vinblastine and Dacarbazine Plus Consolidation Radiotherapy of Residual Nodal Masses for Frontline Treatment in Older Adults With Advanced Stage Classic Hodgkin Lymphoma: Improved Outcome in a Multi‐Center Real‐Life Study
Source: Hematol Oncol. 2024 Nov 17;42(6):e70003. doi: 10.1002/hon.70003 (PMC11590052; doi:10.1002/hon.70003)
Supplement: Supplementary file 3 — Figure S2 [file HON-42-e70003-s001.pptx]

## Slide 1
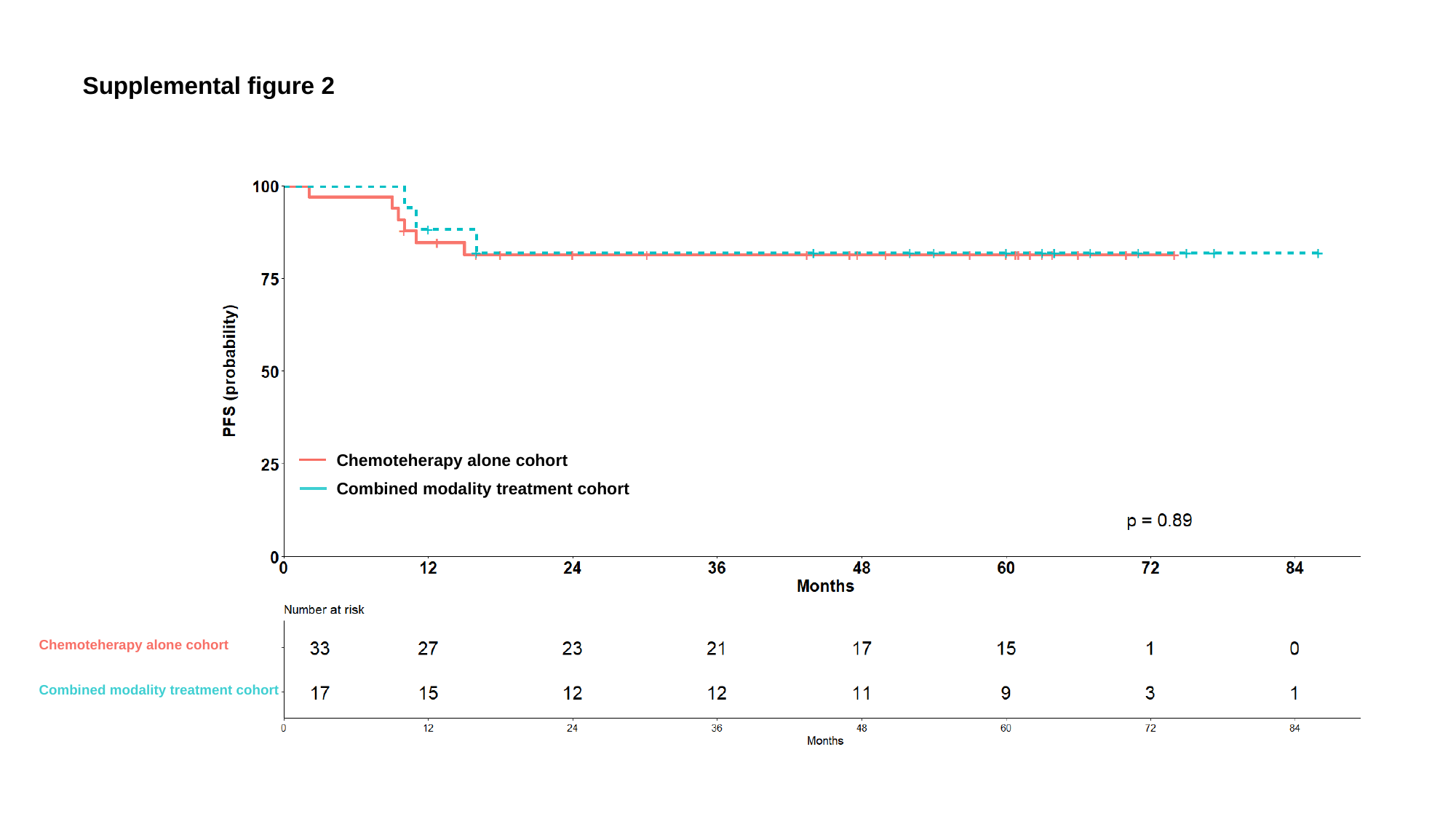

Supplemental figure 2
Chemoteherapy alone cohort
Combined modality treatment cohort
Chemoteherapy alone cohort
Combined modality treatment cohort
