# Supplementary material for: Liposomal Doxorubicin, Vinblastine and Dacarbazine Plus Consolidation Radiotherapy of Residual Nodal Masses for Frontline Treatment in Older Adults With Advanced Stage Classic Hodgkin Lymphoma: Improved Outcome in a Multi‐Center Real‐Life Study
Source: Hematol Oncol. 2024 Nov 17;42(6):e70003. doi: 10.1002/hon.70003 (PMC11590052; doi:10.1002/hon.70003)
Supplement: Supplementary file 4 [file HON-42-e70003-s003.odt]

Supplemental file

Supplemental methods

Data collection

All diagnoses of c-HL were made according to the World Health Organization Lymphoma Classification and confirmed by centralized pathology review by expert hematopathologists applying pathologic criteria current at the time of diagnosis.

Demographic data, disease characteristics, ECOG PS, laboratory values, type of frontline therapy including chemotherapy and/or radiotherapy, response to chemotherapy and/or radiotherapy, date of progression, type of salvage regimen, and date of death or time of last follow‐up were retrieved and collected from the medical records or database. 

Patients were excluded from the analysis if they had concomitant major illnesses at baseline (such as creatinine clearance <30 ml/min, serum transaminases more than three times the normal value, total bilirubin >3.4 mg/dl, absolute neutrophil count <500/mm3, hemoglobin level <9 g/dl and platelet count <75 000/mm3). Myocardial infarction represented an exclusion criterion only if diagnosed within 3 months prior to MVD start. 

All necessary approvals were obtained from our ethic committee (approval protocol number: 65/2024) and ClinicalTrials.gov (number, NCT06235047). The study was undertaken in accordance with the Declaration of Helsinki. All patients provided written informed consent for personal data analysis for research purposes.

Supportive care

Antimicrobials drugs were routinely administered for each patient as follows: trimethropin-sulfamethoxazole at 960 (160 + 800) mg orally every 12 hours for 2 days a week and acyclovir at 800 mg orally daily from the start of chemotherapy until one month after the last cycle. Patients received methylprednisolone at 200 mg i.v. and diphenhydramine at 50 mg i.v., and allopurinol at 300 mg orally (plus hyper-hydration) in every course. 

FDG-PET assessment  

FDG-PET examinations were conducted at staging, end-of-treatment (EoT) (after six MVD cycles ± radiotherapy, as scheduled) and thereafter every three  

months for the first two years, and every six months for the next three years, as previously described26-29. All patients underwent simultaneously whole-body FDG-PET/CT with a combined in-line system (Discovery LS; GE Medical Systems, Milwaukee, WI) that integrates a four-detector row spiral CT (Lightspeed Ultra; GE Medical Systems) with a PET scanner (Discovery LS8; GE Medical Systems). FDG was produced in on-site cyclotron and chemistry facilities. A dose of 5.3 ± 1 MBq/kg of FDG was i.v. injected 60 ± 10 min before imaging. PET scans were carried out from the midbrain to the upper thigh after an 8-h fast, with two-dimensional (2D) emission scans of 4 min per bed position. Images (128 x 128 matrix) were obtained with an iterative ordered subset expectation maximization (OSEM). The OSEM algorithm was applied to ratio sinograms using attenuation-weighted iterative reconstruction (two iterations, 28 subsets) and subsequent smoothing with a Hanning filter. Comparable axial resolutions (full width at half maximum 5.45 mm) were obtained according to the National Electrical Manufacturers Association NU 2001 test procedure. Unenhanced low-dose CT for segmented attenuation correction was carried out in each patient; immediately after, PET scans were acquired covering the same field of view as the CT. FDG-PET and CT data sets were viewed in fused mode using a special fusion workstation. A panel of three nuclear medicine experts (C. Mainolfi, R. Fonti, and S. Del Vecchio) reviewed all scans alongside baseline scans to verify abnormal uptakes in residual or new lymph nodes, or extra-nodal sites. FDG-PET results were reported according to the DS score using visual assessment followed by quantitative verification as already described18,19. In particular, DS scores were assigned as follows: score 1, no residual uptake; score 2, uptake ≤mediastinum; score 3, uptake >mediastinum but ≤liver; score 4, uptake moderately >liver (up to twice the maximal standardized uptake value [SUVmax] in a large region of normal liver); score 5, uptake markedly increased than liver (more than twice the SUVmax in a large region of normal liver) and/or new lesions. At the end of the scheduled program (MVD ± irradiation), negative FDG-PET scans were defined as DS ≤3 score (complete metabolic response), and positive FDG-PET scans (including residual disease with partial metabolic response, or no response with stable disease or progressive disease) were defined as DS 4 and 5 scores18,19. 

Cardiology assessment 

Patients routinely underwent a full cardiologic examination, 2D echocardiography, and speckle tracking echocardiography (STE) at baseline, interim, EoT and within six months from the end of all antineoplastic treatments, according to the guidelines of the Task Force for Cancer Treatments and Cardiovascular Toxicity of the European Society of Cardiology (ESC)5-7. Every echocardiographic examination was preceded by electrocardiogram recording, blood pressure measurement, a full clinical evaluation and a full history that included lifestyle (diet, activity, and smoking habits), family history of cardiac disease, coexistent illnesses, and ongoing therapies, and previous diseases and therapies. Standard transthoracic 2D echocardiographic examinations were performed using a Philips iE33 ultrasound machine (Philips Healthcare, Andover, MA). Images were obtained using a 3.4 MHz transducer with patients in left lateral decubitus position. Following ESC guidelines6,41, standard subxiphoid, apical, and parasternal windows were visualized to acquire 2D images of the cardiac chambers, color, and pulsed- and continuous-wave Doppler measurements. Additional 2D echocardiographic cine loops, using frame rates between 50 and 70 fps, from four-, two-, and three-chamber views were acquired for subsequent offline speckle tracking-derived analysis using a commercially available vendor-independent strain software (Epsilon, Echolnsight, Milwaukee, WI), for STE investigation. The analysis was based on an 18-segment model and GLS was obtained from the mean of the values of the 18 segments. Regional strain values from the six basal, six midventricular, and six apical segments of the left ventricle were averaged to obtain three regional longitudinal strain values (basal, midventricular and apical, respectively). Longitudinal strain analysis allows for evaluation of shortening of a specific portion of the heart obtained by tracking the endocardial border of the left ventricle compared with its original length; it is expressed as a negative percentage. This evaluation is important to estimate myocardial deformation, defined as peak longitudinal systolic strain and expressed as percentage of the longitudinal shortening of systole compared with diastole. A worsening in strain is defined by a less negative number or a smaller deformation in the longitudinal axis, while an improvement is defined by a higher negative number (in its absolute value) or a greater deformation in the same axis, referred to a given segment. Clinical cardiologist experts in echocardiography (G. Esposito, CG. Tocchetti, R. Esposito, M. Prastaro, and C. Santoro) analyzed each study for standard echocardiography and strain measurements. The cut-off values of normality for GLS and LVEF are -20% and 50%, respectively, according to the guidelines of ESC.

Statistical assessment

A predefined study period had to be reached to be representative of a survival report based on adequate (not too short) follow-up. The period of time used in the study was adjusted to provide this result. We consequently acquired over the past decade consistent detailed information on consecutive elderly patients with c-HL in stage III/IV. The primary objective of the study was to evaluate in this setting whether the frontline therapy without bleomycin by using NPLD-containing regimen, i.e., MyocetTM, with its pharmacokinetic and pharmacodynamic advantages in terms of efficacy and safety, followed by consolidation radiotherapy of RNMs, could improve 5-year OS and PFS. Other objectives were EoT response by using FDG-PET scans interpreted with the DS 5-point scoring system18,19, hematologic and extra-hematologic toxicity (according to the National Cancer Institute Common Terminology Criteria for Adverse Events version 5.0), and feasibility (arbitrarily defined as ≤10% of patients receiving <85% of the planned treatment). The dose intensity was defined as: (total dose given/planned)/(cycle days observed/planned).

OS was defined as the time from the date of day 1 of MVD ﬁrst dose to death from any cause (event) or last follow-up visit (censoring). PFS was defined as the time from day 1 of MVD ﬁrst dose to a non-complete response after MVD ± irradiation, i.e., positive FDG-PET scan (DS score ≥4) following the scheduled anticancer treatment (event), relapse at any time (event), death from any cause (event) or last follow-up visit (censoring). The OS and PFS curves were estimated by the Kaplan–Meier method. Patient and disease characteristics, response rate, toxicity and safety data were reported descriptively as number and percentage, or as median and range. For the statistical analysis, the evaluated factors included age stratification (years: 60-69 vs. 70-79 vs. ≥80), B symptoms, LNMs, nodal and/or extra-nodal sites involved, stage IV, and international prognostic score (IPS) risk group at baseline.

Supplemental results

Patient characteristics

Ten patients were excluded due to other frontline chemotherapy regimen (5 patients received ABVD), prior or concurrent lymphoproliferative disease (2 patients excluded due to chronic lymphocytic leukemia/small lymphocytic lymphoma), ECOG PS 4 (at the time of anti-lymphomatous therapy start, 2 patients were completely disabled and confined to bed, thus making it impossible to administer the chemotherapy in outpatient), and insufficient information (for one patient).

As comorbidities, over two-third of patients (80%) had at least two of the traditional cardiac risk factors: age >61 years (n= 40), hypertension (n= 38), hyperlipidemia (n= 35), diabetes mellitus (n= 30), tobacco use (n= 20), obesity (n= 10), and/or history of heart disease, i.e., coronary artery disease (n= 10), atrial fibrillation (n= 8) and heart transplanted for cardiomyopathies (n= 1). In addition, chronic obstructive pulmonary disease was present in 10 patients (20%).

Supplemental figures: Legends

# Figure 1. Overall survival (OS) in the chemotherapy alone cohort and combined modality treatment cohort.

# Five-year OS for the 33 patients receiving chemotherapy alone (MVD) and the 17 patients receiving combined modality treatment (MVD + radiation). Figure also show number of events and number at risk during follow-up.

# Figure 2. Progression free survival (PFS) in the chemotherapy alone cohort and combined modality treatment cohort.

# Five-year PFS for the 33 patients receiving chemotherapy alone (MVD) and the 17 patients receiving combined modality treatment (MVD + radiation). Figure also show number of events and number at risk during follow-up.
